# Pin1-mediated Sp1 phosphorylation by CDK1 increases Sp1 stability and decreases its DNA-binding activity during mitosis

**DOI:** 10.1093/nar/gku1145

**Published:** 2014-11-14

**Authors:** Hang-Che Yang, Jian-Ying Chuang, Wen-Yih Jeng, Chia-I Liu, Andrew H.-J. Wang, Pei-Jung Lu, Wen-Chang Chang, Jan-Jong Hung

**Affiliations:** 1 Institute of Bioinformatics and Biosignal Transduction, College of Bioscience and Biotechnology, National Cheng Kung University, Tainan 701, Taiwan; 2 The PhD Program for Neural Regenerative Medicine, College of Medical Science and Technology, Taipei Medical University, Taipei 110, Taiwan; 3 Center for Bioscience and Biotechnology, National Cheng Kung University, Tainan 701, Taiwan; 4 Core Facilities for Protein Structural Analysis, Academia Sinica, Taipei 115, Taiwan; 5 School of Medical Laboratory Science and Biotechnology, Taipei Medical University, Taipei 110, Taiwan; 6 Institute of Biological Chemistry, Academia Sinica, Taipei 115, Taiwan; 7 Institute of Clinical Medicine, College of Medicine, National Cheng Kung University, 138 Sheng-Li Road, Tainan 70403, Taiwan; 8 Graduate Institute of Medical Sciences, College of Medicine, and Center for Neurotrauma and Neuroregeneration, Taipei Medical University, Taipei 110, Taiwan; 9 Department of Pharmacology, College of Medicine, National Cheng Kung University, Tainan 701, Taiwan; 10 Center for Infectious Disease and Signal Transduction Research, National Cheng Kung University, Tainan 701, Taiwan

## Abstract

We have shown that Sp1 phosphorylation at Thr739 decreases its DNA-binding activity. In this study, we found that phosphorylation of Sp1 at Thr739 alone is necessary, but not sufficient for the inhibition of its DNA-binding activity during mitosis. We demonstrated that Pin1 could be recruited to the Thr739(p)-Pro motif of Sp1 to modulate the interaction between phospho-Sp1 and CDK1, thereby facilitating CDK1-mediated phosphorylation of Sp1 at Ser720, Thr723 and Thr737 during mitosis. Loss of the C-terminal end of Sp1 (amino acids 741-785) significantly increased Sp1 phosphorylation, implying that the C-terminus inhibits CDK1-mediated Sp1 phosphorylation. Binding analysis of Sp1 peptides to Pin1 by isothermal titration calorimetry indicated that Pin1 interacts with Thr739(p)-Sp1 peptide but not with Thr739-Sp1 peptide. X-ray crystallography data showed that the Thr739(p)-Sp1 peptide occupies the active site of Pin1. Increased Sp1 phosphorylation by CDK1 during mitosis not only stabilized Sp1 levels by decreasing interaction with ubiquitin E3-ligase RNF4 but also caused Sp1 to move out of the chromosomes completely by decreasing its DNA-binding activity, thereby facilitating cell cycle progression. Thus, Pin1-mediated conformational changes in the C-terminal region of Sp1 are critical for increased CDK1-mediated Sp1 phosphorylation to facilitate cell cycle progression during mitosis.

## INTRODUCTION

The transcription factor Sp1 plays an important role in regulating the expression of genes involved in many cellular processes by binding to the promoter regions of its target genes. Sp1 binds specifically to GC-rich promoter elements via three C_2_H_2_-type zinc fingers in its C-terminal region and regulates the transcriptional activity of target genes by using two major glutamine-rich transactivation domains localized in its N-terminal region (1). Recent studies showed that the DNA-binding affinity, transactivational activity and protein stability of Sp1 might be regulated by posttranslational modifications including glycosylation, ubiquitination, sumoylation, acetylation and phosphorylation (1–7). Phosphorylation is one of the most studied of the Sp1 modifications, particularly with respect to its role in the expression of genes during the interphase (8,9). For example, Sp1 serine or threonine residues are phosphorylated by different kinases, including DNA-dependent protein kinase, protein kinase A and protein kinase C-ζ, and these phosphorylations cause an increase in the transcriptional activity of Sp1 by increasing its binding to DNA (6,9–12). On the other hand, some kinases can inhibit Sp1 function. During terminal liver differentiation, for example, casein kinase II modifies Thr579 on Sp1 and down-regulates its DNA-binding activity (9,13).

Previous studies indicated that Sp1 accumulates in most types of cancer cells (1,7,14–16). Our recent study showed that Sp1 is modified by c-Jun NH_2_-terminal protein kinase 1 (JNK1) in mitosis, phospho-Sp1 increases its protein stability, and that its presence in cancer cells is higher than that in primary noncancerous cells, indicating that phosphorylation of Sp1 plays an important role for its stability during mitosis (3). Sp1 is known to be displaced from the chromatin and remains stable in mitotic cancer cells, and to be reserved for daughter cells, thus apparently facilitating a quick start to the execution of cell growth (3,17). Our previous findings showed that Sp1 phosphorylation at Thr739 not only increases its protein stability but also decreases its DNA-binding activity during mitosis, thereby benefiting cell cycle progression (18). However, whether phosphorylation of Sp1 at Thr739 is essential for the inhibition of its DNA-binding activity remains unclear.

Pin1 is a peptidyl-prolyl isomerase, which has been shown to be highly expressed in several cancers including prostate, breast, lung and colon cancers (19–22). Pin1 binds specifically phosphorylated Ser/Thr-Pro motifs and promotes *cis-trans* isomerization of proteins, resulting in the regulation of the stability, subcellular localization, interaction, phosphorylation status and the catalytic activity of its client protein (20,23–26). Pin1 contains an N-terminal WW domain and a C-terminal isomerase domain (27,28). The WW domain of Pin1 binds the pSer/Thr-Pro motifs of client proteins to enable the Pin1 isomerase domain to cause a *cis*-to-*trans* conformational change in the prolyl bond of the clients (28,29). Several important proteins such as AMPK, BPGAP1 and BNIP-H/Caytaxin have been reported to interact with Pin1, thereby affecting tumor progression and neuronal differentiation (30–33). A recent study showed that Pin1 is involved in the phosphorylation of SEPT9 by cyclin-dependent kinase 1 (CDK1), which helps to complete the cytokinesis during cell cycle progression (34). We recently showed that CDK1 phosphorylates Sp1 during mitosis. However, the role of Pin1 in relation to Sp1 remains unclear. In fact, several drugs designed to inhibit Pin1 are currently in clinical trial for treating cancers (35–39). However, the specific target or client of Pin1 in cancer cells and the client's importance in cancer remain to be explored.

Mitosis in vertebrates is triggered by CDK1. In the G2 stage, cyclin B1 is accumulated in the cytoplasm, forms a complex with CDK1 and is then phosphorylated at Thr14 and Thr15, causing the inactivation (40). During the late G2 phase, CDK1 activation begins with its dephosphorylation by phosphatase CDC25, and most of the cyclin B1/CDK1 complexes are translocated rapidly from the cytoplasm into the nucleus while the nuclear envelope breaks down (40). Our recent study demonstrated that CDK1 is a novel kinase that phosphorylates Sp1 at Thr739 during mitosis and, consequently, represses the DNA-binding ability of Sp1. Moreover, myosin/F-actin was found to interact with phospho-Sp1 resulting in its displacement from the chromatin, which is required for chromosome packaging during mitosis (18). Therefore, the complete phosphorylation of Sp1 is essential for cell cycle progression of cancer cells. In this study, we found that Sp1 phosphorylation at Thr739 during mitosis was necessary, but not sufficient for the movement of Sp1 out of the chromosomes. In addition to the phosphorylation of C-terminal of Sp1 by CDK1, a Pin1-dependent highly phosphorylation of Sp1 by CDK1 is critical for maintaining its protein stability and for its complete movement out of the chromosomes, which is important for cell cycle progression.

## MATERIALS AND METHODS

### Cell culture and transfection

HeLa cells and mouse embryonic fibroblasts (MEFs) were cultured in DMEM containing 10% of FBS, 100μg/ml streptomycin sulfate and 100 units/ml penicillin G sodium at 37°C, 5% CO_2._ For transfection of GFP-Sp1, GFP-Sp1-T739A, GFP-Sp1-T739D, GFP-Sp1 (1-740), GFP-Sp1-4A, GFP-Sp1-4D, HA-Sp1, HA-Sp1-4A (S720A, T723A, T737A and T739A), HA-Sp1-4D (S720D, T723D, T737D and T739D), pGL2, pGL2-p21, Sp1 shRNA, or Pin1 shRNA, 2 μg of plasmids was incubated with 2 μl of Lipofectamine in 200 μl of OPTI-MEM for 30 min in room temperature. After incubation, the mixture of plasmids and Lipofectamine in OPTI-MEM was added into 2 ml of OPTI-MEM. The cells were incubated with the mixture of plasmids and Lipofectamine for 6 h. The cells were then incubated in fresh Dulbecco's modified Eagle's medium (DMEM) for 18 or 36 h. The cells were collected for immunoprecipitation or immunoblotting.

### Western blotting

Cell lysates or immunoprecipitation samples were fractionated by sodium dodecyl sulfate-polyacrylamide gel electrophoresis (SDS-PAGE) and transferred to polyvinylidene fluoride (PVDF) membrane using a transfer apparatus according to the manufacturer's protocols (Bio-Rad). After incubation with 3% non-fat milk or 2% bovine serum albumin in Tris-buffered Saline with Tween 20 buffer (TBST buffer, 10 mM Tris-HCl pH 8.0, 150 mM NaCl and 0.5% Tween 20) for 1 h, membranes were incubated with antibodies at 4°C overnight. Membranes were washed four times for 5 min and incubated with the horseradish-peroxidase-conjugated anti-mouse or anti-rabbit antibodies with 1:3000 dilutions for 1 h. After incubation, membranes were washed with TBST four times for 5 min and developed with the enhanced chemiluminescence (ECL) system (Millipore) according to the manufacturer protocols.

### Immunoprecipitation

Cell lysates were prepared by using mRIPA buffer (50 mM Tris-HCl, pH 7.8, 150 mM NaCl, 5 mM ethylenediaminetetraacetic acid (EDTA), 0.1% Triton-X100, 0.05% NP-40). The lysates were incubated with anti-Sp1 (1:500), anti-CDK1 (1:200), anti-Pin1 (1:250), anti-HA tag (1:250), or anti-GFP tag (1:200) antibodies at 4°C for 1 h. After 1 h incubation, the mixture was incubated with Protein A or G Sepharose agarose beads at 4°C for 1 h. The beads were collected and washed three times by mRIPA buffer. The beads were added 2X electrophoresis sample buffer and analysed by immunoblotting.

### Cell synchronization

HeLa cells were blocked at the G1/S boundary with 2 mM thymidine (Sigma-Aldrich) for 18 h. The cells were then washed three times with phosphate buffered saline (PBS) and incubated with fresh medium. And 10 h after release, the cells were re-plated onto 6-cm plates with 2 mM thymidine and re-incubated for 16 h. Plates were then washed three times with PBS and fresh medium was added. The time point, corresponding to the G1/S transition, was defined as 0 h. The cells were collected after different time intervals (0, 3, 6, 8, 10, 12 and 14 h). Equal amounts of proteins from these cell extracts were analysed using immunoblotting. For another kind of cell synchronization, HeLa cells were treated by 45 ng/ml nocodazole for 16 h. Mitotic cells were collected by mechanical shake-off. For the nocodazole release, the synchronized cells were then washed three times with PBS and re-plated in fresh medium. The released cells were then collected after different time intervals and then analysed using immunoprecipitation and immunoblotting.

### Immunofluorescence and confocal microscopy

The cells were fixed with 4% paraformaldehyde (Sigma-Aldrich) in PBS according to a method described previously (3). Immunostaining was conducted with primary antibodies such as anti-Sp1, anti-GFP tag (Santa Cruz Biotechnology, Santa Cruz, CA), or anti-Pin1 (Calbiochem, San Diego, CA) antibodies. The cells were then treated with Alexa Fluor 488-conjugated goat anti-mouse or rabbit immunoglobulin G (IgG) and Alexa Fluor 568-conjugated goat anti-mouse or rabbit IgG polyclonal antibodies (Jackson ImmunoResearch Laboratories Inc., West Grove, PA). Finally, the cells were mounted in 90% glycerol containing 4'-6-diamidino-2-phenylindole (DAPI) (Invitrogen), and examined using a confocal laser-scanning microscope (FluoView^TM^ FV 1000; Olympus, Melville, NY) or immunofluorescence microscope (Personal DV Applied Precision, Issaquah, WA). The images were analysed with *softWoRx* software (Applied Precision Inc.).

### *In vitro* CDK1/cyclin B1 kinase assay

For the *in vitro* phosphorylation analysis, the GST-Sp1(619-785), GST-Sp1(619-785)-T739A, GST-Sp1(619-785)-T739D, GST-Sp1(619-785)-P6, GST-Sp1(619-785)-Z12, GST-Sp1(619-715) or GST-Sp1(619-740) were purified from *Escherichia coli* BL21 (DE3). These different Sp1 proteins and active CDK1/cyclin B1 (New England Biolabs, Beverly, MA) were used to examine Sp1 phosphorylation *in vitro*. Each reaction (20 μl) contained 1 μg of purified Sp1, 50 ng of active CDK1/cyclin B1, 2 μCi of [γ-^32^P] ATP (GE Healthcare Life Sciences), 1 mM ATP (New England Biolabs) and 2 ml of 10× kinase buffer containing 500 mM HEPES pH 7.4, 10 mM MgCl_2_, 1 mM EGTA and 1 mM dithiothreitol. The phosphorylation reactions were incubated at 30°C for 15 min. After the incubation, one-half of the reaction was added to 10 μl of 2×electrophoresis sample buffer, which was then heated to 95°C for 5 min. Proteins in the mixtures were immediately separated using SDS-polyacrylamide gel electrophoresis (PAGE).

### GST-pull-down assay

GST, GST-Pin1, GST-Pin1 (W34A) or His-Pin1 and GST-Sp1(619-785), GST-Sp1(619-785)-T739A, GST-Sp1(619-785)-T739D at a concentration of 40 ng/ml in 500 μl of NaCl, EDTA, Tris-HCl and NP-40 buffer (NETN buffer, 20 mM Tris-HCl pH 8.0, 100mM NaCl, 1 mM EDTA and 0.5% NP-40), containing 10 μg/ml each of leupeptin, aprotinin and 4-(2-aminoethyl)-benzenesulfonyl fluoride, was added to 30 μl of glutathione–Sepharose 4B beads (GE Healthcare Life Sciences). The mixture was incubated with shaking for 1 h at 4°C. The beads were washed three times in NETN buffer and added to the interphase or mitotic cell extract. The reaction mixture was incubated on ice for 1 h. The beads were then washed in NETN buffer. Proteins were eluted by sample buffer and separated by SDS- PAGE.

### Fluorescence-activated cell sorting (FACS) analysis

The cells washed by cold PBS and then fixed in 70% alcohol or 4% paraformaldehyde at 4°C for 2 h. After fixation, the cells washed by cold PBS and treated with 0.1% TritonX-100, 10μg/ml RNase and 50 μg/ml propidium iodide for 1 h. The cells were analysed by flow cytometer (Cell Lab Quanta™ SC; Beckman Coulter or FACSCalibur; BD Biosciences).

### DNA affinity precipitation assay (DAPA)

Cell lysate incubated with 1 μg of binding probes in 500 μl binding of buffer (2 mM MgCl_2_, 60 mM KCl, 20 mM Tris-HCl pH 7.8, 5% glycerol, 0.5 mM EDTA and 0.02% NP-40). After incubation for 1 h at 4°C, the mixture was incubated with 30 μl of streptavidin-agarose beads (GE Healthcare Life Sciences) for 1 h at 4°C. The mixture was washed three times by binding buffer. The proteins in the beads were eluted by 2X sample buffer and analysed by SDS-PAGE and immunoblotting. The Sp1-binding probe 5′-CCCGCCTCCTTGAGGCGGGCCCGGGCGGGGCGG-3′, localized from −82 to −50 bp within the promoter of p21^WAF1/CIP1^, was biotinylated at 5′ termini and then annealed with complementary strands.

### Protein expression, purification and crystallization

Owing to the R14A mutant of Pin1 exhibits higher stability and no interference with Pin1 phosphopeptide-binding (WW domain) or catalytic activity (PPIase domain) (41), the R14A mutant of Pin1 was used widely instead of wild-type Pin1 in many previously structural studies. Additionally, two other point mutants of Pin1, K63A and C113A, which are PPIase-activity-deficient, were generated and used in the crystallographic experiments (42). For the crystallization and the thermodynamic parameters of Pin1 and Sp1 peptide binding analysis, protein expression and crystallization of Pin1 was performed in slightly modified as described previously (41). In brief, the gene for Pin1 was cloned into pET28a with an N-terminal His_12_-tag and a thrombin cleavage site. R14A, R14A/K63A and R14A/C113A mutants of Pin1 were generated using the PCR-based QuikChange site-directed mutagenesis kit (Agilent). The correct construct was transformed into *E. coli* BL-21 (DE3) competent cells for protein expression. Transformed BL21 (DE3) bacteria were grown in Luria Bertani (LB) medium containing 50 mg/ml kanamycin at 30°C. Bacteria were induced with 1 mM isopropyl *β*-D-thiogalactoside (IPTG) overnight at 20°C. Pin1 proteins were purified by Ni^2+^-nitrilotriacetic acid affinity chromatography, digested with thrombin and further purified by second run of Ni^2+^-nitrilotriacetic acid affinity chromatography. The protein was prepared at 10–20 mg/ml in 10mM HEPES pH 7.5 containing 100 mM NaCl and 1 mM DTT for crystallization. Crystals were grown from a drop composed of 1 ml of protein solution and 1 ml of reservoir solution consisting of 2.0–2.5 M (NH_4_)_2_SO_4_, 1–2% (w/v) PEG 400 and 1–2 mM DTT in 0.1M HEPES buffer pH 7.5 equilibrated at 4°C against 0.5 ml of reservoir solution using the hanging-drop vapor-diffusion method. Crystals of Pin1 in complex with Sp1 peptides were obtained in co-crystallization experiments with the Pin1 protein pre-mixed with 10-fold Sp1 peptide and incubated at 4°C for 4 h before crystallization.

### Diffraction data collection

All crystals were flash-cooled to 100 K in a stream of cold nitrogen prior to data collection. Prior to flash-cooling, the crystals were soaked briefly in a cryoprotectant solution. The cryoprotectant solution consisted of 2.5 M (NH_4_)_2_SO_4_, 2% (w/v) PEG 400 and 30% glycerol. X-ray diffraction data were collected on SPXF beamlines BL13B1, BL13C1 and BL15A1 at the National Synchrotron Radiation Research Center (NSRRC), Taiwan. The electron density was calculated by Refmac5 (43). The electron density is shown by using the Coot software (44).

### Isothermal titration calorimetry (ITC) analysis

Thermodynamic parameters of Pin1 and Sp1 peptide binding were determined using a Micro-Cal iTC200 (GE Healthcare Life Sciences). All samples were filtered with 0.22-mm cutoff filters (Millipore). The buffer for Pin1 and Sp1 peptide solutions consisted of 10 mM HEPES pH 7.5, 100 mM NaCl and 1 mM DTT. A 900 mM solution of Sp1 peptide was directly titrated into a solution of 60 mM Pin1 in 200-ml aliquots. The experiments were performed at 25°C. Data were then analysed using the software origin.

### Protein identification by mass spectrometry analysis

The interacting proteins of CDK1 during mitosis in MEF and MEF^Pin1-/−^ cells were identified by immunoprecipitation with anti-CDK1 antibodies in mitotic cell lysates and analysed by silver staining. The protein band increased in MEFs on the silver staining gel was excised for identification by mass spectrometry analysis. The in-gel digestion, mass spectrometry analysis and database search were performed as described previously (45).

### Bioinformatic analysis

The proteins network of Fn1/NuMA/EWSR1/RBMX (hnRNP-G) was created by MetaCore™. The interaction related with cell cycle (GO: 0007049) and regulation of mitosis (GO: 0007088) were be selected and analysed.

### Statistical analysis

The statistical analysis of differences of western blotting, reporter assay, or flow cytometry was calculated with Student's t test. Data are representative of three independent experiments and as mean ± s.e.m. *P* values less than 0.05 were considered statistically significant.

## RESULTS

### Pin1 recruitment facilitates highly phosphorylation of Sp1 by CDK1

**Figure 1.**
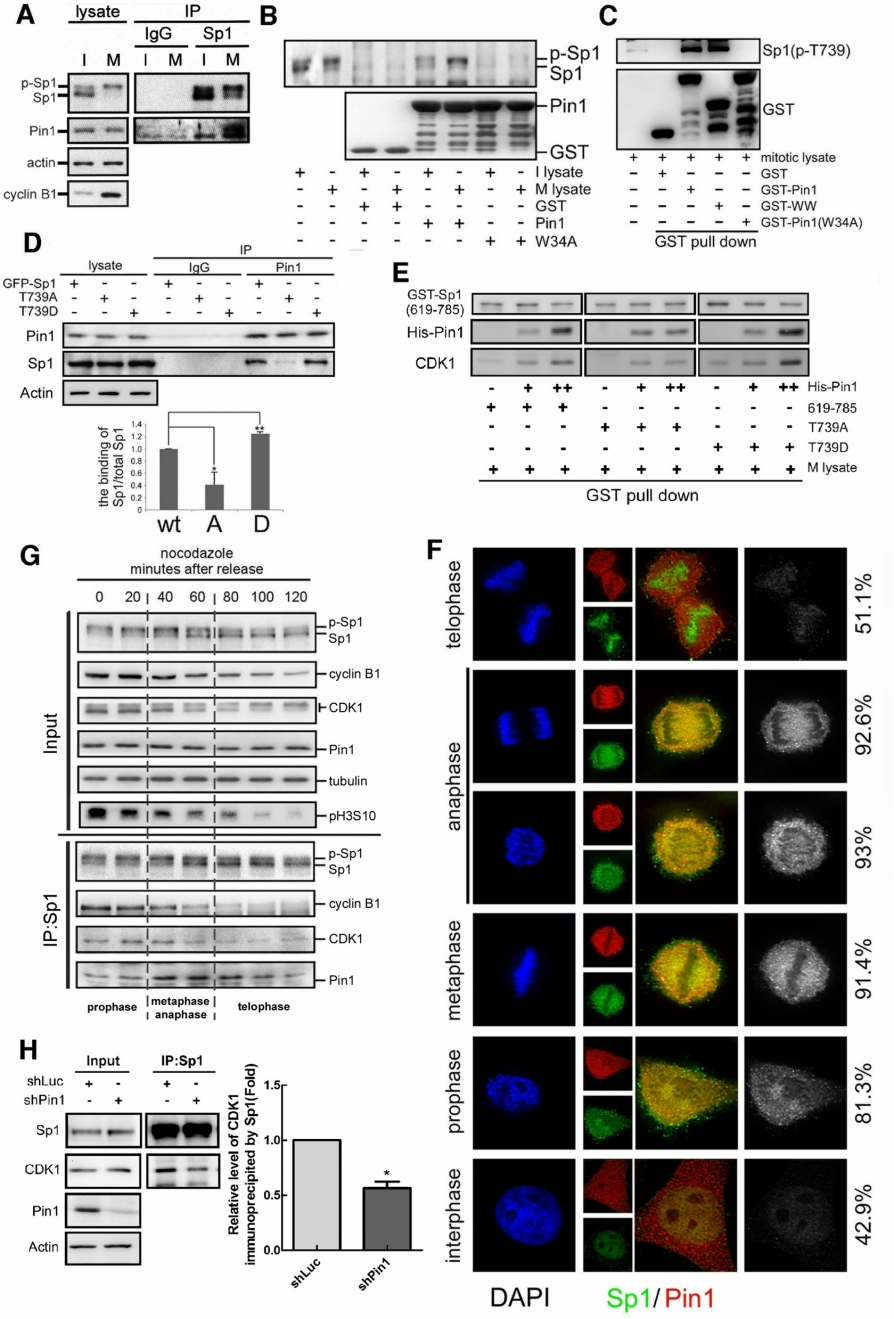
Pin1 interacts with Thr739(p)-Sp1. (**A**) Interphase (I) and mitotic (M) HeLa lysates were harvested for immunoprecipitation assay with anti-Sp1 antibodies. IP samples were analysed with western blotting with antibodies against Sp1, Pin1, actin and cyclin B1. (**B**) GST-Pin1 and its mutant, W34A, were synthesized in *E. coli* and purified for pull down assay with cellular lysates from interphase and mitotic stages. Recruited samples were analysed by western blotting with antibodies against Sp1 and GST. I lysate: interphase cell lysate; M lysate: mitotic cell lysate. (**C**) GST-Pin1 and its mutant, W34A and WW-domain, were synthesized in *E. coli* and purified for pull down assay with cellular lysates from mitotic stage. Recruited samples were analysed by western blotting with antibodies against phospho-Sp1 (T739) and GST. (**D**) GFP-Sp1 and its mutants, T739A and T739D, were expressed in HeLa cells. Mitotic cell lysates were collected for immunoprecipitation (IP) assay with anti-Pin1 antibodies. IP samples were analysed by western blotting with antibodies against Pin1, Sp1 and actin. The quantitative data of Pin1-interacted Sp1 are represented as mean ± s.e.m.(*P<0.05 and **P<0.01) (**E**) GST-Sp1 (619–785) was expressed in *E. coli* and purified for pull down assay with HeLa cell lysate with His-Pin1 expression. Recruited samples were analysed by western blotting with antibodies against GST, His-tag and CDK1. M lysate: mitotic cell lysate. (**F**) The co-localization of Sp1 and Pin1 in the various cell cycle stages was studied by immunofluorescence (IF) assay with antibodies against Sp1 (green) and Pin1 (red). Chromosomes are marked by DAPI (blue) and the co-localized ratio was shown in the right. (**G**) HeLa cells were synchronized at mitotic stage by nocodazole treatment, then harvested the lysates at different time courses after nocodazole release for immunoprecipitation assay with anti-Sp1 antibodies. IP samples were analysed by western blotting with antibodies against Sp1, cyclin B1, pH3S10, CDK1, Pin1 and Tubulin. (**H**) Pin1 was knocked down by shPin1 in HeLa cells. Cell lysates were harvested for immunoprecipitation assay with anti-Sp1 antibodies. IP samples were analysed by western blotting with antibodies against Sp1, CDK1, actin and Pin1. The quantitative data are represented as mean ± s.e.m. (*P<0.05).

We previously showed that phosphorylation of Sp1 at Thr739 is important for modulating its protein stability and DNA-binding activity (3,18). Since this phosphorylation site is a Thr(P)-Pro motif, we speculated that Pin1 interacted with Sp1. In this study, we found that Pin1 interacted with Sp1 in mitotic cells, but not in interphase cells (Figure 1A). Using recombinant wild-type Pin1 and a Pin1 mutated in the recognition domain, Pin1-W34A, in pull down assays with cell lysates of the interphase and mitotic cells, we found that Pin1, but not Pin1-W34A, interacted with mitotic Sp1, indicating that Pin1, via its recognition domain, interacts with Sp1 specifically during mitosis (Figure 1B). Since most of the Sp1 is phosphorylated during mitosis, we found that GST-Pin1 and its recognition motif, GST-WW, interacted with phospho-Sp1(T739) (Figure 1C). To study the significance of the phosphorylation of Sp1 at Thr739 with respect to its interaction with Pin1, Sp1 or truncated versions of Sp1, carrying different mutations, namely, Sp1-T739A and Sp1-T739D, were constructed to examine their interaction with Pin1 (Figure 1D and E). The phosphorylation site mutated Sp1, Sp1-T739A showed decreased interaction with Pin1, but the mimic phosphorylated Sp1, Sp1-T739D, showed increased interaction with GST-Pin1, indicating that Sp1 phosphorylation at Thr739 is critical for its interaction with Pin1. Then, we studied the interaction between Sp1 and Pin1 in various sub-mitotic stages (Figure 1F and G). Within 20 min of nocodazole release, high levels of cyclin B1 and H3S10 phosphorylation suggested that cells stayed in the prophase, and that Sp1 interacted with Pin1 partially (81.3%). Within 20–60 min, cyclin B1 and H3S10 phosphorylation were partially decreased, indicating that cells stayed in the metaphase and anaphase (46). Sp1 strongly interacted with Pin1 in these two phases (91.4% and 93% respectively; Figure 1F and G). After 60 min, the level of cyclin B1 and H3S10 phosphorylation were decreased and cells entered into the telophase, and Sp1 lost the interaction with Pin1. We recently showed that CDK1 phosphorylates Sp1 during mitosis (18). Here, we found that silencing Pin1 decreased the interaction between Sp1 and CDK1, implying that Pin1 may be involved in the phosphorylation of Sp1 by CDK1 (Figure 1H). In summary, these results demonstrate that Pin1 specifically interacts with phospho-Sp1 during mitosis and may play a role in the phosphorylation of Sp1 by CDK1.

### Pin1 modulates conformational changes at the C-terminal end of Sp1 to increase highly phosphorylation of Sp1 by CDK1

**Figure 2.**
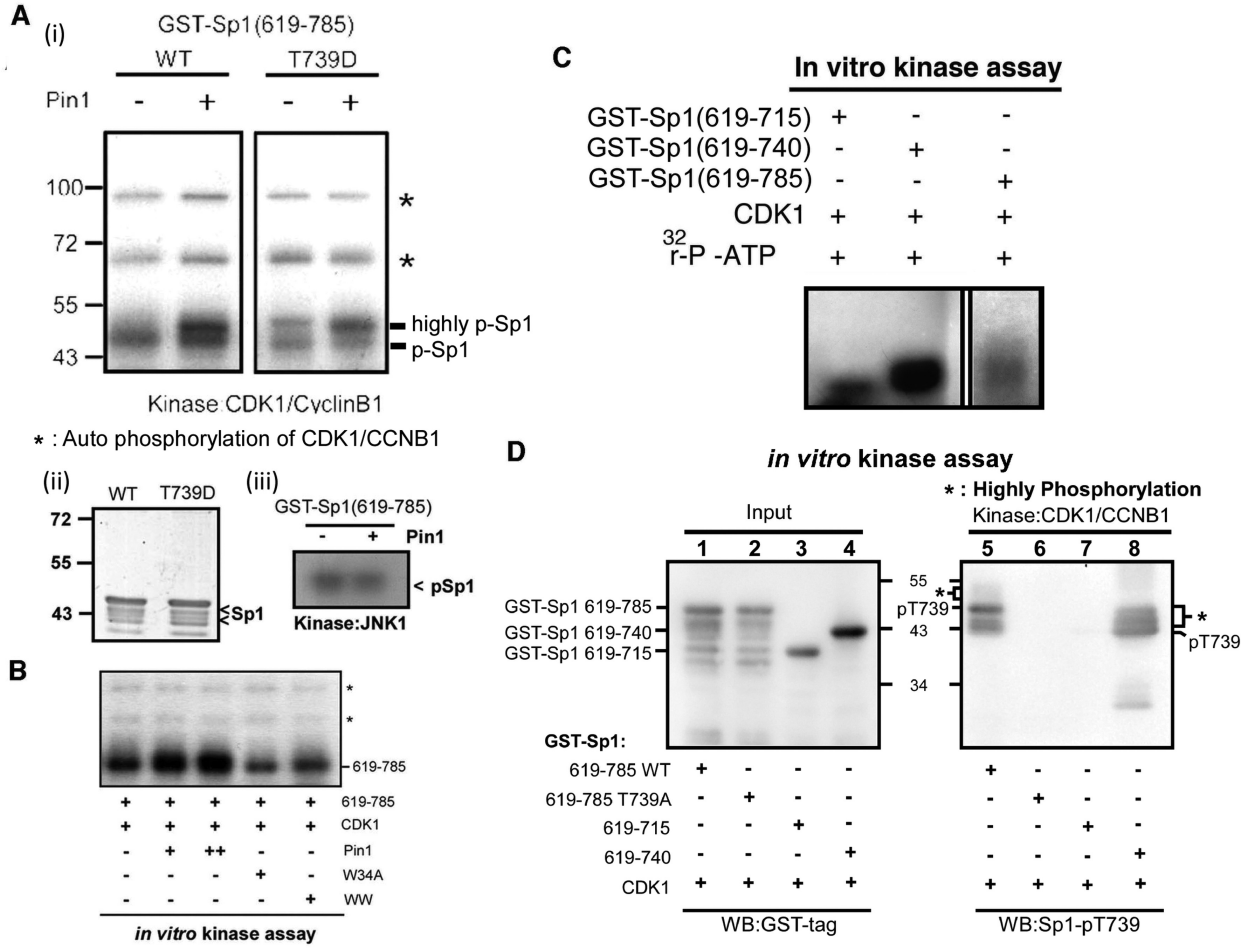
Pin1 increases Sp1 phosphorylation. (**A**) GST-Sp1 (619–785) and T739D were expressed in *E. coli* and purified for *in vitro* kinase assay with CDK1 (i) and JNK1 (iii) in the absence or presence of Pin1, and Sp1 level as an internal control (ii). (**B**) GST-Sp1 (619–785) was expressed in *E. coli* and purified for *in vitro* kinase assay with CDK1 with Pin1, W34A or WW-domain. *: autophosphorylated CDK1/cyclin B1 (**C**) GST-Sp1 (619–715), GST-Sp1 (619–740) and GST-Sp1 (619–785) were expressed in *E. coli* and purified for *in vitro* kinase assay with CDK1. (**D**) Various recombinant proteins as indicated were expressed in *E. coli* and purified for *in vitro* kinase assay with CDK1. The samples were analysed by western blotting with antibodies against Sp1 phosphorylation at T739 and GST tag. *: band shift of highly phosphorylated Sp1.

A truncated versions of Sp1, corresponding to its wild-type C-terminal region and its mimic phosphorylation proteins, GST-Sp1(619-785)-WT and GST-Sp1(619-785)-T739D, were expressed as substrates for the *in vitro* kinase assay in the presence or absence of Pin1 (Figure 2A). Sp1 was strongly phosphorylated by CDK1 in the presence of Pin1 (Figure 2A-i). Although Sp1 is known to be phosphorylated by JNK1 at Thr739 (3), this phosphorylation remained unaffected, implying that the Pin1-mediated highly phosphorylation of Sp1 by CDK1 is specific (Figure 2A-iii). Although we know that the WW-domain alone can interact with Thr739-Sp1 (Figure 1C), it was of interest to determine whether the interaction between Sp1 and Pin1 was sufficient for highly phosphorylation of Sp1 by CDK1. Wild-type Pin1 and its mutants, Pin1-WW and Pin1-W34A, were used in the *in vitro* kinase assay with CDK1 (Figure 2B). We found that wild-type Pin1, but not Pin1-W34A and Pin1-WW, increased the signal of CDK1-mediated Sp1 phosphorylation in a dose dependent manner, indicating that not only the interaction between Sp1 and Pin1 but also the isomerase activity of Pin1, is essential for the increased phosphorylation of Sp1 by CDK1. Since the isomerase activity of Pin1 alters the *cis*/*trans* states of its substrate, Pin1 may modulate the C-terminal conformation of Sp1, thereby affecting the interaction between Sp1 and CDK1. Ablation of the C-terminus (amino acids 741-785) of Sp1 significantly increased the phosphorylation signal, implying that the C-terminus inhibits Sp1 phosphorylation by CDK1 (Figure 2C). In addition, GST-Sp1(619-715), as a substrate of CDK1, lost most of the signal, implying that the phosphorylation residue(s) are localized within the region 715–740 (Figure 2C). When GST-Sp1 (619–785), GST-Sp1 (619-785)-T739A, GST-Sp1 (619–715) and GST-Sp1 (619–740) were used in the *in vitro* kinase assay with CDK1, GST-Sp1 (619–785) was phosphorylated by CDK1, albeit to a lesser extent (Figure 2D, lane 1). No signal was detected for GST-Sp1 (619–785)-T739A and GST-Sp1 (619–715), suggesting that T739 contributes to the phosphorylation by CDK1 (Figure 2D, lanes 6 and 7). Finally, an increased phosphorylation signal was obtained when the C-terminus of Sp1 (741–785 residues) was ablated, indicating that the C-terminal end of Sp1 inhibits increased phosphorylation (Figure 2D, lane 8).

**Figure 3.**
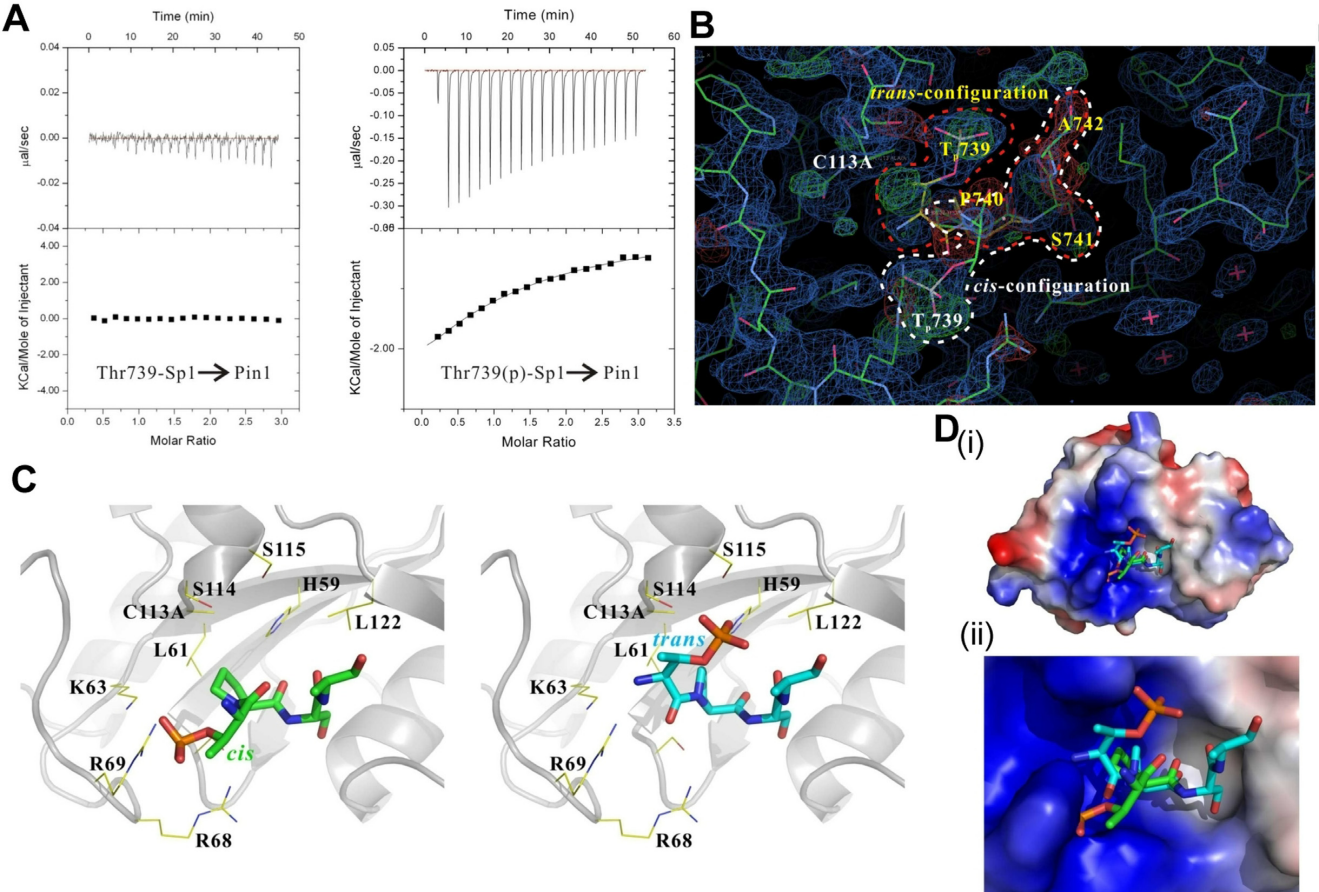
Pin1 recruitment alters the conformation of C-terminal Sp1. (**A**) The binding analyses of Thr739-Sp1 (left panel) and Thr739(p)-Sp1 (right panel) peptides to Pin1 were determined by ITC. Representative plots from an ITC experiment are shown with raw data in the upper panel and curve fit in the lower panel. The Thr739-Sp1 or Thr739(p)-Sp1 peptides were titrated into the reaction cell containing Pin1 protein. Thermodynamic values obtained from the curve fit of Thr739(p)-Sp1 peptide titrated with Pin1 are: ΔS = -5.0 cal/mol/K, ΔH = -6.8 ± 2.2 kcal/mol, *K*a = 7.4 ± 1.4 × 10^3^ M^−1^, N = 0.93 ± 0.25. N is the stoichiometry of bound Thr739(p)-Sp1 peptide per Pin1 protein. (**B**) View of the electron density map around the active site of Pin1 bound the Thr739(p)-Sp1 peptide presenting to two configurations. The *cis*- and *trans*-configurations of Thr739(p)-Sp1 peptide were indicated by white and red dashed lines, respectively. 0.5 occupancy for each configuration of Thr739(p)-Sp1 peptide model was used for the electron density calculation. The 2Fo–Fc electron density is shown in blue color, contoured at 1σ. (**C**) The *cis*- and *trans*-configurations of Thr739(p)-Sp1 peptide and the surrounding residues in the active site of Pin1 structure. (**D**) Electrostatic surface representations of Pin1 bound with Thr739(p)-Sp1 peptide in two configurations. Overall structure (i) and a close-up view of the active-site pocket of Pin1 (ii). The two configurations of Thr739(p)-Sp1 peptide are shown as stick models. Positive charges on the electrostatic surfaces are drawn in blue and negative charges in red.

To confirm that Pin1 indeed affects the C-terminal conformation of Sp1, Pin1 was purified and oligopeptides of the region 735–745 of Sp1, with or without phosphate at Thr739, were synthesized for ITC and X-ray crystallography experiments (Figure 3). The binding analysis of Sp1 peptides to Pin1 by ITC demonstrated that Pin1 could interact with the Thr739(p)-Sp1 peptide but not with the Thr739-Sp1 peptide (Figure 3A). The ITC results showed that Pin1 interacted with the Thr739(p)-Sp1 peptide with a dissociation constant (*K*_d_) of ∼50 mM. In addition, owing to the R14A mutant of Pin1 exhibits higher stability and no interference with Pin1 phosphopeptide-binding (WW domain) or catalytic activity (PPIase domain) (41), the R14A mutant of Pin1 was widely used instead of wild-type Pin1 in numerous structural studies previously. Furthermore, two other point mutants of Pin1, K63A and C113A, which are PPIase-activity-deficient, were generated and used in the crystallographic experiments (42). X-ray crystallography data showed that the Thr739(p)-Sp1 peptide, as substrate of Pin1, was bound to the active site of Pin1. Finally, one configuration of the electron density map of the Thr739(p)-Sp1 peptide was observed in the Pin1 R14A mutant, while two configurations of the electron density map of the Thr739(p)-Sp1 peptide were observed in the Pin1 R14A/C113A mutant with ablated isomerase activity (Figure 3B and D). These results indicate that Pin1 can recruit the Thr739-phosphorylated Sp1 to alter the C-terminal conformation of Sp1 via the conversion of the *cis*/*trans* peptide bond between Thr739(p) and Pro740.

### Pin1-mediated increase in the highly phosphorylation of Sp1 by CDK1 is important for the inhibition of the DNA-binding activity of Sp1 during mitosis

**Figure 4.**
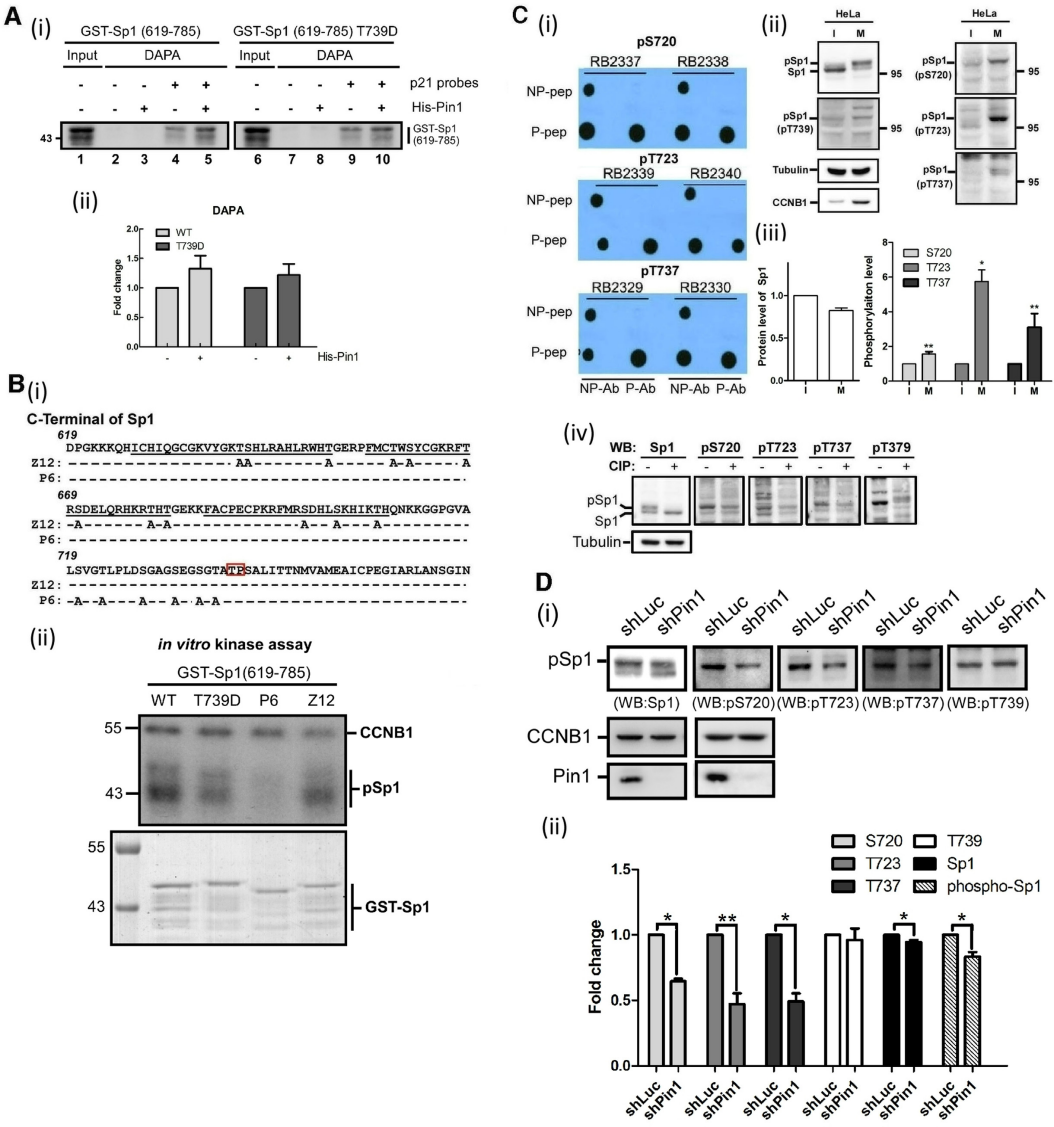
Highly phosphorylation of Sp1 in mitosis. (**A**) GST-Sp1(619–785) and GST-Sp1(619–785)T739D were expressed in *E. coli* and then purified for studying the DNA-binding activity with DAPA assay (i). The level of probes-bound Sp1 was quantified from four independent experiments with Student's t test. There was no significance (ii) (**B**) Various threonine and serine in the C-terminus of Sp1 were mutated into alanine as indicated, Z12 and P6 (i). Wild type (WT), T739D, P6 and Z12 were expressed in *E. coli* and purified for *in vitro* kinase assay with CDK1 (ii). (**C**) Six residues, threonine or serine, within P6 were changed to glutamate individually, then to synthesize the specific phospho-Sp1 antibodies for western blot or dot blot with HeLa cell lysate from interphase (I) or mitotic (M) cells (i and ii), and CIP treated cell lysates (iv). The quantitative data are represented as mean ± s.e.m. Only statistically significant *P* values are shown (*P<0.05 and **P<0.01) (iii) (**D**) Pin1 was knocked down by shRNA in HeLa cells, and then harvested for western blot with antibodies against Sp1 phosphorylation at Ser720, Thr723, Thr737 and T739. Data are representative of three independent experiments and as mean ± s.e.m (*P<0.05 and **P<0.01).

Here, we purified the GST-Sp1 (619–785) and its mimic phosphorylation form, GST-Sp1 (619–785)-T739D, to study the DNA-binding activity of Sp1 (Figure 4A). No significant difference in the DNA-binding signal was observed between GST-Sp1 (619–785) and its phosphorylation form, implying that Sp1 phosphorylation at Thr739 alone may not be sufficient to influence its DNA-binding activity.

Based on these results, we then examined the amino acid residue(s) of Sp1 that are phosphorylated by CDK1 during mitosis (Figure 4B, C and Supplementary Figure S1). To this end, we mutated all serine and threonine residues located between amino acids 619–739 of Sp1 (mutants Z12 and P6), and performed the *in vitro* kinase assay with CDK1 (Figure 4B). For the Z12 mutant with all 12 serine and threonine residues (between amino acids 619–710) mutated, the phosphorylation signal remained unchanged. However, the signal was nearly abolished in the P6 mutant with six serine and threonine residues (between amino acids 719–739) mutated, implying that the phosphorylation residues are located within this region. Therefore, we synthesized phospho-peptides to design antibodies that can recognize these six phospho-residues endogenously. First, these antibodies were tested for their specificity in recognizing of the peptides and phospho-peptides (Figure 4C-i). All antibodies recognized the phospho-peptides, but not the peptides, suggesting that the antibodies were specific. The six antibodies recognized only Ser720, Thr723 and Thr737 in HeLa cell lysates, indicating that Sp1 can be phosphorylated at Ser720, Thr723 and Thr737 during mitosis (Figure 4C-ii, -iii and Supplementary Figure S1). To confirm the specificity, Sp1 was knocked down by shRNA, or the mitotic cell lysate was treated by CIP, which in turn decreased the signals exhibited by the antibodies (Figure 4C-iv and Supplementary Figure S2). Finally, the phosphorylation signals were also decreased in cells with Pin1 knock-down (Figure 4D), suggesting that Pin1 is indeed involved in the highly phosphorylation of Sp1 during mitosis. Phosphorylation of Sp1 at Thr739 is critical for the inhibition of its DNA-binding activity during mitosis (18).

### C-terminus of Sp1 is involved in its stability and DNA-binding activity

**Figure 5.**
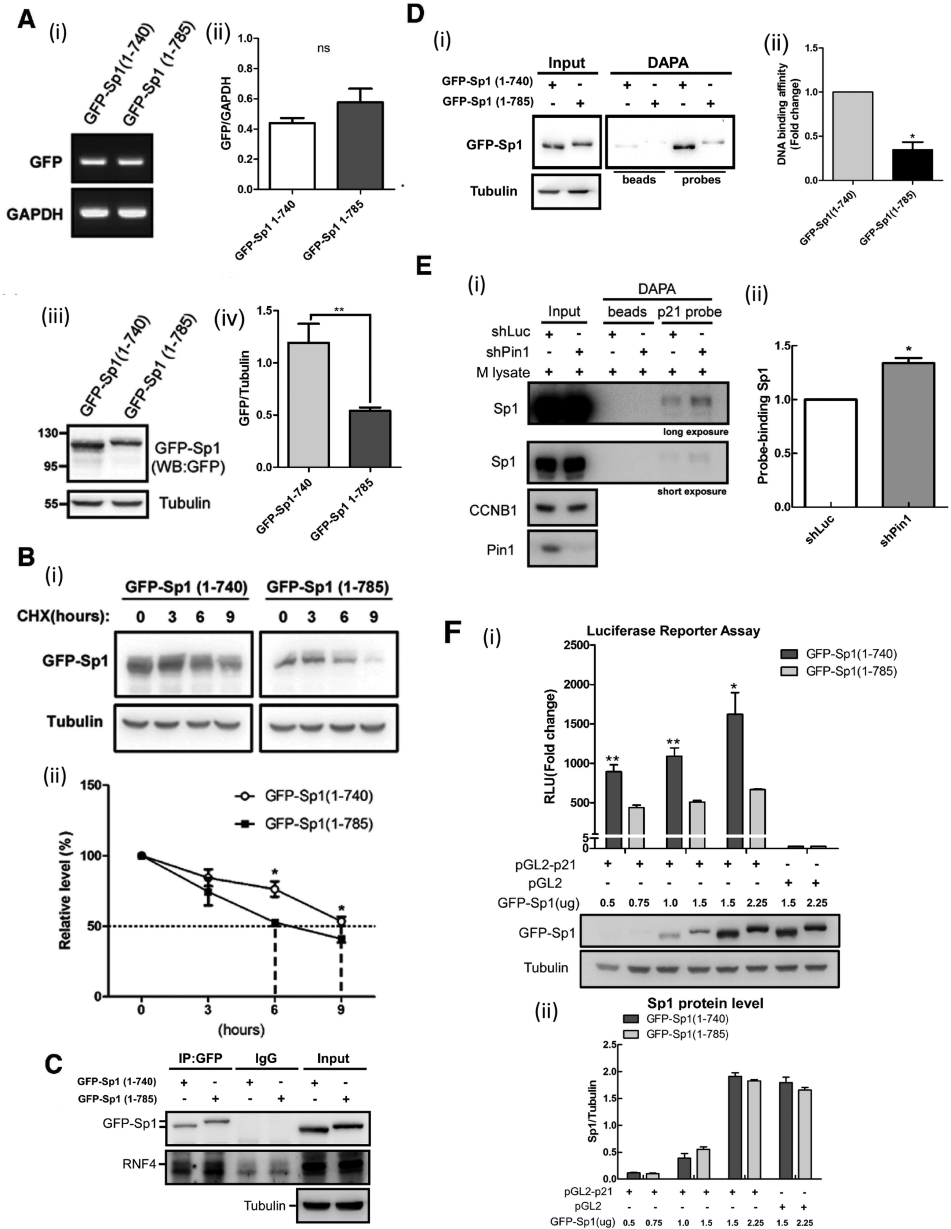
C-terminus of Sp1 involves in its protein stability and DNA-binding activity. (**A**) Total RNA was extracted and cell lysates was harvested from HeLa cells with GFP-Sp1(1–740) or GFP-Sp1(1–785) overexpression for RT-PCR with primer recognised GFP mRNA (i and ii), and western blotting with anti-GFP antibodies (iii and iv). The quantitative data of protein or RNA level are represented as mean ± s.e.m. (*P<0.05, ns: no significant) (**B**) Cell lysates were collected from cycloheximide-treated HeLa cells with GFP-Sp1 (1–740) or GFP-Sp1 (1–785) overexpression for western blotting with anti-GFP and anti-Tubulin antibodies (i). The quantitative data of Sp1 protein are represented as mean ± s.e.m. (*P<0.05) (ii). (**C**) Cell lysates were collected from HeLa cells with overexpression of GFP-Sp1 (1–740) or GFP-Sp1 (1–785) individually for immunoprecipitation assay with anti-GFP antibodies. IP samples were analysed by immunoblotting with antibodies against GFP, RNF4 and Tubulin. Nuclear extracts were isolated from HeLa cells with GFP-Sp1 (1–740) or GFP-Sp1 (1–785) overexpression (**D**), or Pin1 knock-down (**E**) for DAPA assay with probe with Sp1-binding elements from p21^CIP/WAP^ promoter. DAPA samples were analysed by immunoblotting with antibodies against GFP, Sp1, CCNB1, Pin1 and Tubulin. The quantitative data are represented as mean ± s.e.m. (*P<0.05). M Lysate: mitotic HeLa cell lysates. (**F**) Equal amount of GFP-Sp1 (1–740) and GFP-Sp1 (1–785) were expressed in the luciferase-expressed HeLa cells for luciferase activity assay. The protein level and luciferase activity were quantified and analysed by Student's t test. The quantitative data are represented as mean ± s.e.m. (*P<0.05 and **P<0.01).

**Figure 6.**
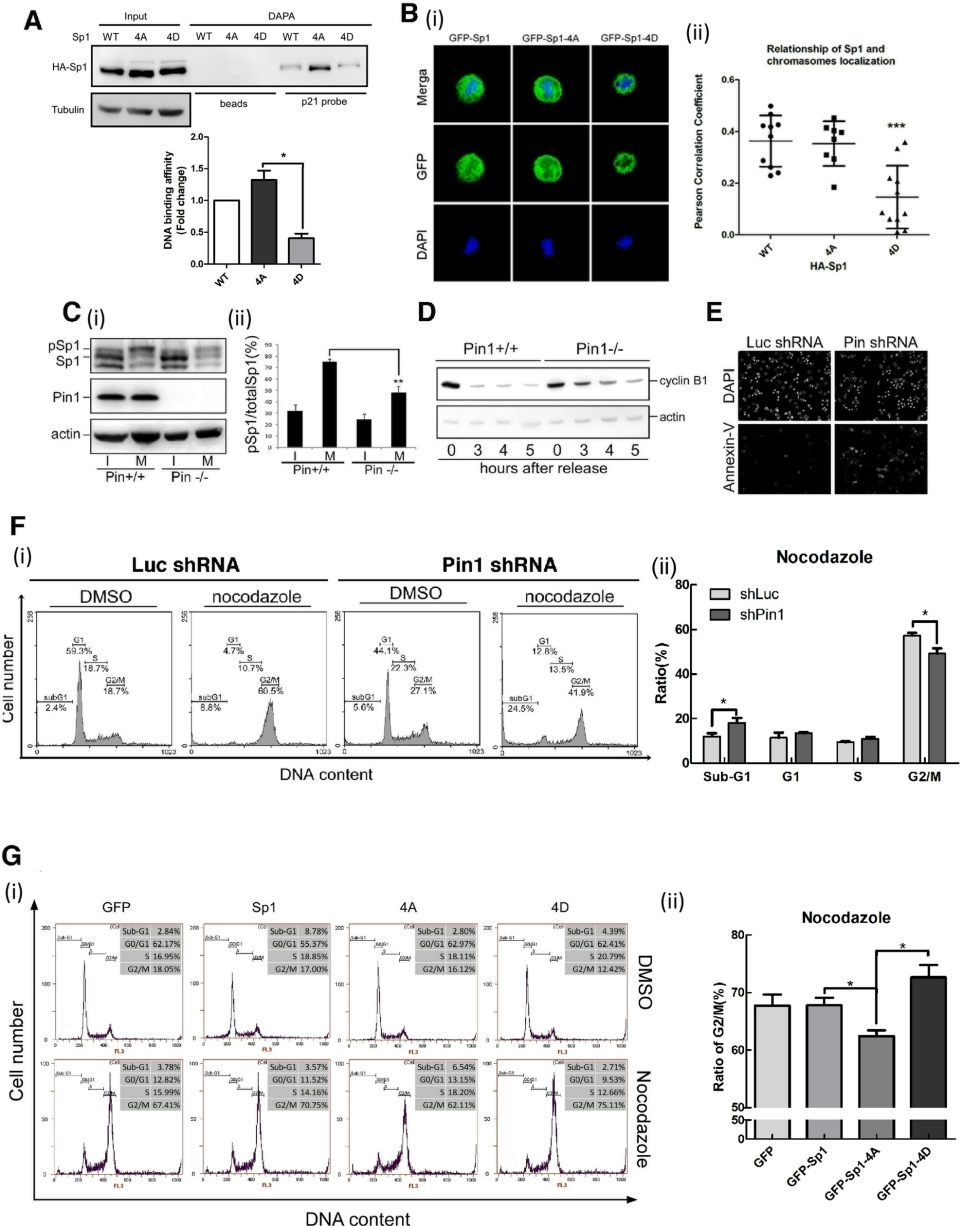
Pin1-mediated highly phosphorylation of Sp1is important for cell cycle progression. (**A**) HA-Sp1, HA-Sp1-4A(S720A, T723A, T737A and T739A) and HA-Sp1-4D(S720D, T723D, T737D and T739D) were expressed in HeLa cells for 24 h individually. Mitotic extract was harvested and incubated with p21^CIP/WAP^ probe for DAPA assay. The DAPA samples were analysed by SDS-PAGE and immunobloting with anti-HA and anti-Tubulin antibodes. Data were quantified after three independent experiments and expressed as mean ± s.e.m. (**B**) HeLa cells were grown on coverslips in DMEM and expressed GFP-Sp1, GFP-Sp1-4A and GFP-Sp1-4D for 24 h individually. Cells were then synchronized by nocodazole for 16 h. After synchronization, the HeLa cells were fixed using 4% paraformaldehyde, and labeled with anti-Sp1 (green) and anti-GFP (green) antibodies. DNA was stained with DAPI (blue) (i). The ratio of colocalized chromosomes and Sp1 were quantified by *softWoRx* software (Applied Precision Inc.) and calculated by Pearson's correlation coefficient (ii). ***P<0.001 were considered as significantly different. (**C**) MEF^Pin1+/+^ and MEF^Pin1-/-^ cells were synchronized with nocodazole and cell lysates were analysed for immunoblotting by anti-Sp1, anti-actin, or anti-Pin1 antibodies (i). Ratio of phospho-Sp1 was quantified and normalized against total Sp1 from three independent experiments and analysed by t-test for statistical significance (ii). (**D**) Mitotic MEF^Pin1+/+^ and MEF^Pin1-/−^ cells were collected by nocodazole synchronization. After releasing of nocodazole, cells were harvested at different time points as indicated and analysed for immunoblotting with anti-cyclin B1. (**E** and **F**) HeLa cells were transfected with Luc shLuc or shPin1 for 24 h and treated with nocodazole for another 16 h. The cells were fixed for detecting the signal of annexin-V by Immunofluorescence microscopy with Alexa 568-labeled annexin-V or for flow cytometry assay by PI staining. (**G**) HeLa cells were transfected with GFP-Sp1-WT, 4A and 4D for 24 h and treated with nocodazole for another 16 h. The cells were fixed for flow cytometry assay by PI staining. The quantitative data are represented as mean ± s.e.m. (*P<0.05).

**Figure 7.**
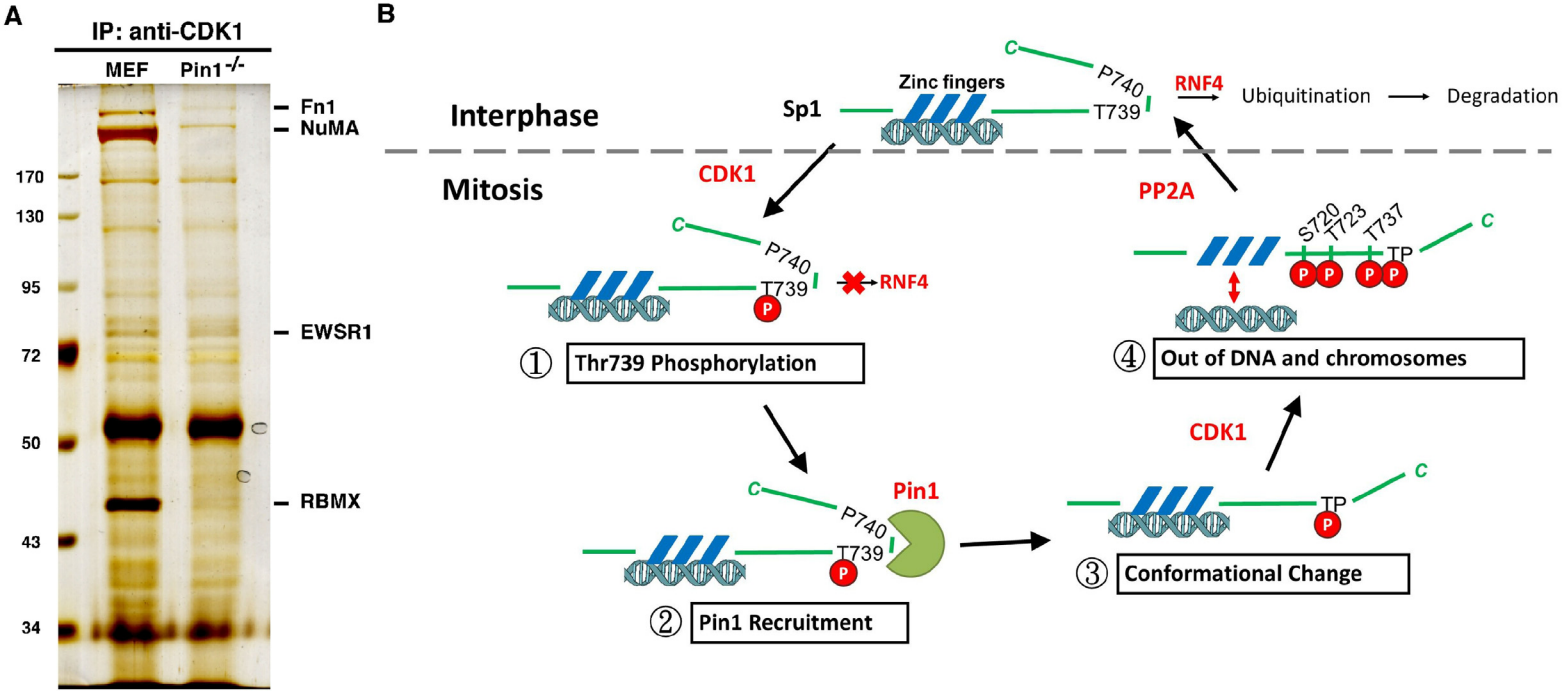
Pin1 knockout decreases the interaction between CDK1 and its interacted proteins. (**A**) Cell lysate were harvested from MEF and MEF^Pin1-/-^ cells for immunoprecipitation assay with anti-CDK1 antibodies. (**B**) The hypothesis of Pin-1-mediated highly phosphorylation of Sp1 by CDK1 in mitosis.

We found that the C-terminus of Sp1 was important for highly phosphorylation of Sp1 during mitosis and that Pin1 could regulate the C-terminal conformation of Sp1. We then investigated the role of the C-terminus of Sp1 in the stability and DNA-binding activity of Sp1. Equal amount of plasmids were transfected into HeLa cells to express the same mRNA levels. The protein level of GFP-Sp1 (1–740) was higher than that of GFP-Sp1 (1–785) (Figure 5A-iii and -iv). Loss of the C-terminal end of Sp1 increased protein stability, suggesting that this is involved in the degradation of Sp1 (Figure 5B). RNF4 recruitment is critical for the degradation of Sp1 (47). Here, we found decreased interaction between RNF4 and GFP-Sp1 (1–740) compared to that between RNF4 and full-length Sp1 (1–785), indicating that the C-terminal end of Sp1 (amino acids 741–785) is important for its interaction with RNF4, which in turn enhances Sp1 degradation (Figure 5C). In addition, loss of the C-terminus and Pin1 knock-down increased the DNA-binding activity of Sp1 during mitosis, implying that Pin1 may modulate the C-terminus of Sp1 to regulate the DNA-binding activity of during mitosis (Figure 5D and E). Since the C-terminus increases Sp1 degradation and suppresses its DNA-binding activity, we studied the effect of C-terminus deprivation on down regulation of DNA-binding activity and transcriptional activity of Sp1. Equal amount of GFP-Sp1 (1–740) and GFP-Sp1(1–785) were expressed in HeLa cells to study their effect on the transcriptional activity of p21^CIP/WAP^ (Figure 5F). Compared to the full-length GFP-Sp1 (1–785), GFP-Sp1 (1–740) increased the transcriptional activity of p21^CIP/WAP^, indicating that the C-terminus of Sp1 negatively regulates the expression of p21^CIP/WAP^ by affecting Sp1's DNA-binding activity. We have demonstrated that CDK1 can phosphorylate Sp1 at Ser720, Thr723, Thr737 and Thr739 during mitosis. To study the effects of these phosphorylation residues on the DNA-binding activity of Sp1 and on cell cycle progression, we mutated these four residues to aspartate (D) and showed that this ?mimic phosphorylation mutant?, Sp1–4D, decreased the DNA-binding activity of Sp1 to the p21^CIP/WAP^ promoter. In contrast, when those residues were changed to alanine (A), the Sp1–4A mutant increased its DNA-binding activity to the p21^CIP/WAP^ promoter, suggesting that Pin1-mediated highly phosphorylation of Sp1 by CDK1 inhibits the DNA-binding activity of Sp1 (Figure 6A). We have previously shown that Sp1 leaves the chromosomes during mitosis, thereby facilitating cell cycle progression (18). When we studied the cellular distribution of GFP-Sp1, GFP-Sp1-4A and GFP-Sp1-4D during mitosis, we found that only GFP-Sp1-4D was completely removed from the chromosomes (Figure 6B) We analysed the effect of Pin1 on highly phosphorylation of Sp1 in wild-type MEF and MEF^Pin1-/−^ cells and found that the loss of Pin1 decreased the level of highly phosphorylated Sp1 (Figure 6C, lane 4). After nocodazole release, the wild-type MEFs were in past mitosis stage within 3 h, but the mitosis in MEF^Pin1-/-^ was delayed until 5 h, indicating that Pin1 is involved in the progression of mitosis, possibly by affecting highly phosphorylation of Sp1 (Figure 6D). Inhibition of Sp1 phosphorylation during mitosis is known to induce cell apoptosis (15). Here, Pin1 knock-down also increased the annexin-V signal and sub-G1 percentage (Figure 6E and F). Finally, Sp1 and its mutants, Sp1-4A and Sp1-4D, were overexpressed to study the cell cycle progression. After nocodazole treatment, only 62% of the total Sp1-4A-expressing cells entered mitosis. In contrast, 75% of the total Sp1-4D-expressing cells entered mitosis after nocodazole treatment, implying that the increased highly phosphorylation of Sp1 at these four residues truly contributes to cell cycle progression (Figure 6G). Our results have indicated that the recruitment of Pin1 to Sp1 modulated highly phosphorylation of Sp1 by CDK1. We then examined other CDK1-interacting proteins that may be affected by Pin1. Immunoprecipitation with anti-CDK1 antibodies in wild-type MEF and MEF^Pin1-/−^ cells showed that several proteins such as fibronectin 1 (Fn1), nuclear mitotic apparatus protein (NuMA), Ewing sarcoma breakpoint region 1 (EWSR1), and the RNA-binding motif protein, X chromosome (RBMX), showed decreased interaction with CDK1 in MEF^Pin1-/-^ cells (Figure 7A). Using the bioinformatics tool MetaCore^TM^ to analyse the network of these four proteins, we found that p53 might have a close relationship with the four Pin1-mediated CDK1-interacting proteins (Supplementary Figure S3).

## DISCUSSION

In this study, we have provided evidence regarding the translocation of Sp1 in and out of the chromosomes during cell cycle progression. CDK1 phosphorylates Sp1 at Thr739, thereby recruiting Pin1 to the C-terminal end of Sp1 to alter its conformation, which is critical for highly phosphorylation of Sp1 at Ser720, Thr723 and Thr737, which in turn causes Sp1 to move out of the chromosomes completely (Figure 7B).

Several phosphorylation sites among the 785 amino acids of Sp1 can affect the transcriptional activity of Sp1, positively or negatively, by modulating its DNA-binding affinity, transactivation, or total protein expression, depending on the position of the amino acid or the condition of the cell (6).

We recently found that Sp1 is a mitotic substrate of CDK1/cyclin B1 and is phosphorylated by CDK1/cyclin B1 at Thr739. Phosphorylation of Sp1 decreases its DNA-binding ability and facilitates the process of chromatin condensation during mitosis (18). We previously demonstrated that Thr739-phosphorylated Sp1 begins to move out of the chromosomes during the prophase and moves out of the chromosomes completely in the metaphase. At the end of the telophase and the beginning of the interphase, Sp1 is dephosphorylated by PP2A and move back to the chromosomes. Cancer cells use CDK1 and PP2A to regulate the movement of Sp1 in and out of the chromosomes during the cell cycle. In addition, Sp1 is phosphorylated at Thr668 by casein kinase II (CKII) to decrease its DNA-binding activity. Treatment of K562 cells with okadaic acid increases Sp1 phosphorylation and inhibits its DNA-binding activity, suggesting that steady-state levels of Sp1 phosphorylation are established by a balance between kinase and phosphatase activities (13,48). However, phosphorylation of Sp1 at Thr668, Ser670 and Thr681 by protein kinase C-ζ (PKC-ζ) is required for Sp1-dependent platelet-derived growth factor-D (PDGFD) activation in response to angiotensin II (49). Further studies are needed to understand why phosphorylation of Sp1 in this region has different effects on its transcriptional activity.

In this study, we found that Sp1 phosphorylation at Thr739 during mitosis is necessary, but not sufficient for Sp1 to move out of the chromosomes, leading to the process of chromosomal condensation. Several residues of Sp1, namely, Ser720, Thr723 and Thr737, are phosphorylated by CDK1 in a Pin1-dependent manner. Our data demonstrate that highly phosphorylation of Sp1 and not Thr739 alone is essential for the complete inhibition of the DNA-binding activity of Sp1 during mitosis, which is critical for chromosomal condensation, leading to cell cycle progression. Initially, mimicking the phosphorylation of Sp1 at Thr739 did not alter its DNA-binding activity. However, mutating all four phosphorylation residues, Ser720, Thr723, Thr737 and Thr739, increased the DNA-binding activity of Sp1, and mimicking phosphorylation at these residues decreased its DNA-binding activity and caused Sp1 to move out of the chromosomes completely during mitosis. In addition, our previous study also indicated that Sp1 phosphorylation at Thr739 increases its protein stability by preventing the recruitment of ubiquitin E3-ligase, RNF4 (47). Here, we found that highly phosphorylation of Sp1 also increased its protein stability. Similarly, JNK1-mediated phosphorylation of Sp1 at Thr739 during the interphase increased he stability of Sp1 (3). However, this JNK1-mediated phosphorylation at Thr739 in interphase cannot induce highly phosphorylation of Sp1. In summary, these results indicate that Sp1 phosphorylation at Thr739 alone might be sufficient for increasing the stability of Sp1.

Cyclin-dependent kinase 1 (CDK1) is a highly conserved protein that functions as a serine/threonine kinase, and is a key player in cell cycle progression (50–53). Over 75 proteins, identified as substrates of CDK1 in the budding yeast, are important for cell cycle progression (54–58). Our previous study showed that Sp1 could be phosphorylated by CDK1 at Thr739 to increase its protein stability and decrease its DNA-binding activity. This causes Sp1 to move out of the chromosomes during mitosis, facilitating cell cycle progression (18). Here, we demonstrate a novel mechanism of how CDK1 phosphorylates its substrate, Sp1. Our data show that Pin1 is involved in the highly phosphorylation of Sp1 by CDK1 during mitosis. Pin1 increases the phosphorylation signal by CDK1 and recruits to the Thr739(p)-Pro motif of the C-terminus of Sp1, following which it increases the interaction between Sp1 and CDK1. Finally, Pin1 alters the conformation of the C-terminus of Sp1. This is the first time we have addressed the role of the C-terminus of Sp1 for its protein stability and DNA-binding activity. The protein level of truncated Sp1 (amino acids 1–740) increased when the C-terminus of Sp1 was deleted (Figure 5), indicating that the C-terminus of Sp1 (amino acids 741–785) contributes to the degradation of Sp1. Consistently, our previous study indicated that the C-terminus of Sp1 interacts with RNF4, the E3-ligase of Sp1 (47). Therefore, Sp1 phosphorylation by JNK1 and CDK1 at Thr739 may modulate the C-terminal conformation of Sp1 to inhibit the recruitment of RNF4, thus stabilizing Sp1. However, as indicated in Figure 4D-i, knock-down of Pin1 only slightly decreased the level Sp1. Our previous studies indicated that Thr739, phosphorylated by JNK1, is critical for stabilizing the level of Sp1 (3,47). We found that Pin1 interacted with Thr739(p)-Sp1, leading to highly phosphorylation of Sp1 at the Ser720, Thr723 and Thr737 residues. Based on these findings, we suppose that Sp1 phosphorylation at Thr739 has only partially contributed to Sp1 stability. Therefore, Pin1-mediated highly phosphorylation of Sp1 is critical for the decrease in DNA-binding activity of Sp1, but only partially affects the level of Sp1. In addition, loss of the C-terminus of Sp1 increased its DNA-binding activity to the p21 promoter, indicating that conformational changes in the C-terminus itself may affect the interaction between the three Sp1 DNA-binding domains and the p21^CIP/WAP^ promoter (Figure 5). However, here we found that the conformation of the C-terminal end of Sp1 can be modulated by Pin1 recruitment, which induced highly phosphorylation of Sp1 by CDK1, thereby decreasing its DNA-binding activity during mitosis.

Pin1, as an isomerase, mediates conformational changes of several proteins during mitosis (38,59). Many important proteins have been identified as substrates of Pin1 (42,60–65). CDK1 is essential for regulating cell cycle progression. Sp1 is a critical transcription factor that regulates the expression of a number of genes related to cell proliferation and development. In this comprehensive study on the role of the C-terminal structure of Sp1 for its stability and DNA-binding activity, we have demonstrated, for the first time, an association between Pin1 and CDK1. Since CDK1 phosphorylates many proteins to modulate their functions in mitosis, we believe that Pin1 not only regulates Sp1 conformation but also affects other substrates of CDK1, leading to their highly phosphorylation during mitosis, thereby facilitating cell cycle progression.

We also probed several Pin1-regulated CDK1-interacting proteins such as Fn1, NuMA, EWSR1 and RBMX. Fn1 is involved in cell motility, wound healing, adhesion and maintenance of cell shape (66–70). NuMA with its microtubule-binding ability is involved in the functioning of mitotic centrosomes (71–73). EWSR1 has RNA-binding activity and plays a role in post-transcriptional processing during mRNA splicing (74–77). RBMX is involved in pre- and post-transcriptional processes such as alternative splicing (78–80). By using a bioinformatics tool to analyse the network between cell cycle progression and these proteins, we found that these proteins might be related to the function of p53 in cell cycle progression. However, the functional relationship between Pin1-mediated CDK1-interacting proteins and p53 remains to be clarified. In this study, we demonstrate a novel role of Pin1 in highly phosphorylation of CDK1 substrates. We expect that additional CDK1 substrates may also be highly phosphorylated in a Pin1-dependent manner during mitosis; however, their complete repertoire remains to be investigated.

## SUPPLEMENTARY DATA

Supplementary Data are available at NAR Online.

SUPPLEMENTARY DATA
