# Supplementary material for: Pin1-mediated Sp1 phosphorylation by CDK1 increases Sp1 stability and decreases its DNA-binding activity during mitosis
Source: Nucleic Acids Res. 2014 Nov 14;42(22):13573–87. doi: 10.1093/nar/gku1145 (PMC4267622; doi:10.1093/nar/gku1145)
Supplement: SUPPLEMENTARY DATA [file supp_42_22_13573__index.html]

Pin1-mediated Sp1 phosphorylation by CDK1 increases Sp1 stability and decreases its DNA-binding activity during mitosis — Pin1-mediated Sp1 phosphorylation by CDK1 increases Sp1 stability and decreases its DNA-binding activity during mitosis — SUPPLEMENTARY DATA 

# Pin1-mediated Sp1 phosphorylation by CDK1 increases Sp1 stability and decreases its DNA-binding activity during mitosis

## SUPPLEMENTARY DATA

**Files in this Data Supplement:**

- SUPPLEMENTARY DATA
