# Supplementary material for: Pin1-mediated Sp1 phosphorylation by CDK1 increases Sp1 stability and decreases its DNA-binding activity during mitosis
Source: Nucleic Acids Res. 2014 Nov 14;42(22):13573–87. doi: 10.1093/nar/gku1145 (PMC4267622; doi:10.1093/nar/gku1145)
Supplement: SUPPLEMENTARY DATA [file supp_gku1145_nar-01924-x-2014-File009.pdf]

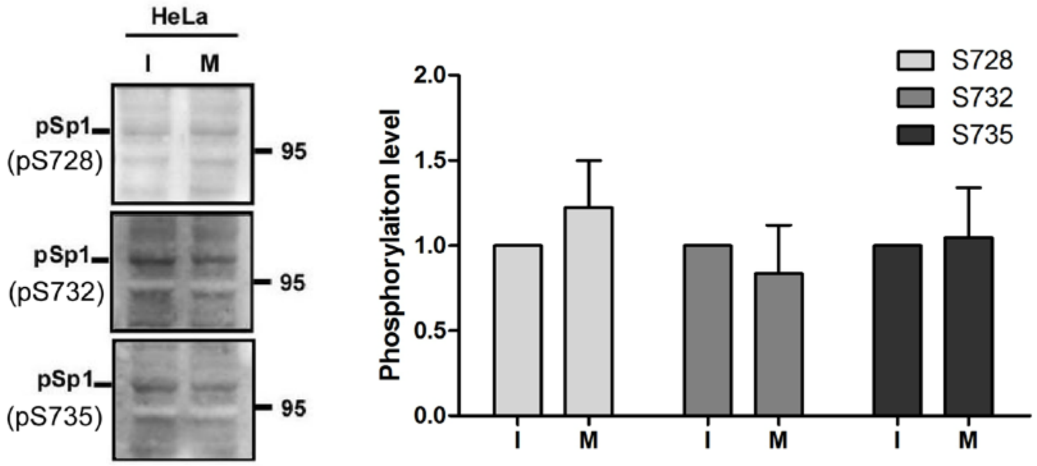

Supplementary Figure 1 HeLa cell lysate from interphase (I) or mitotic (M) cells were collected for Western blot with the anti-phospho-S728, anti-phospho-S732, and anti-phospho-S735 antibodies. Data are representative of three independent experiments and as mean ± s.e.m

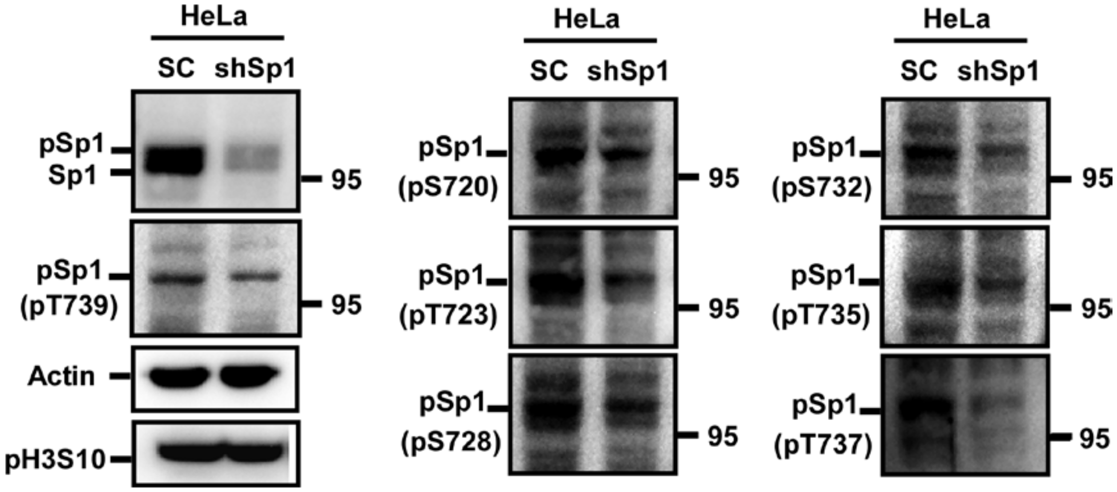

Supplementary Figure 2 Sp1 was knocked down by shRNA in HeLa cells, and the mitotic cell lysates were harvested for Western blot with antibodies against Sp1 phosphorylation at S720, T723, S728, S732, T735, T737, and T739. (SC: Scramble control, shSp1: Sp1 shRNA)

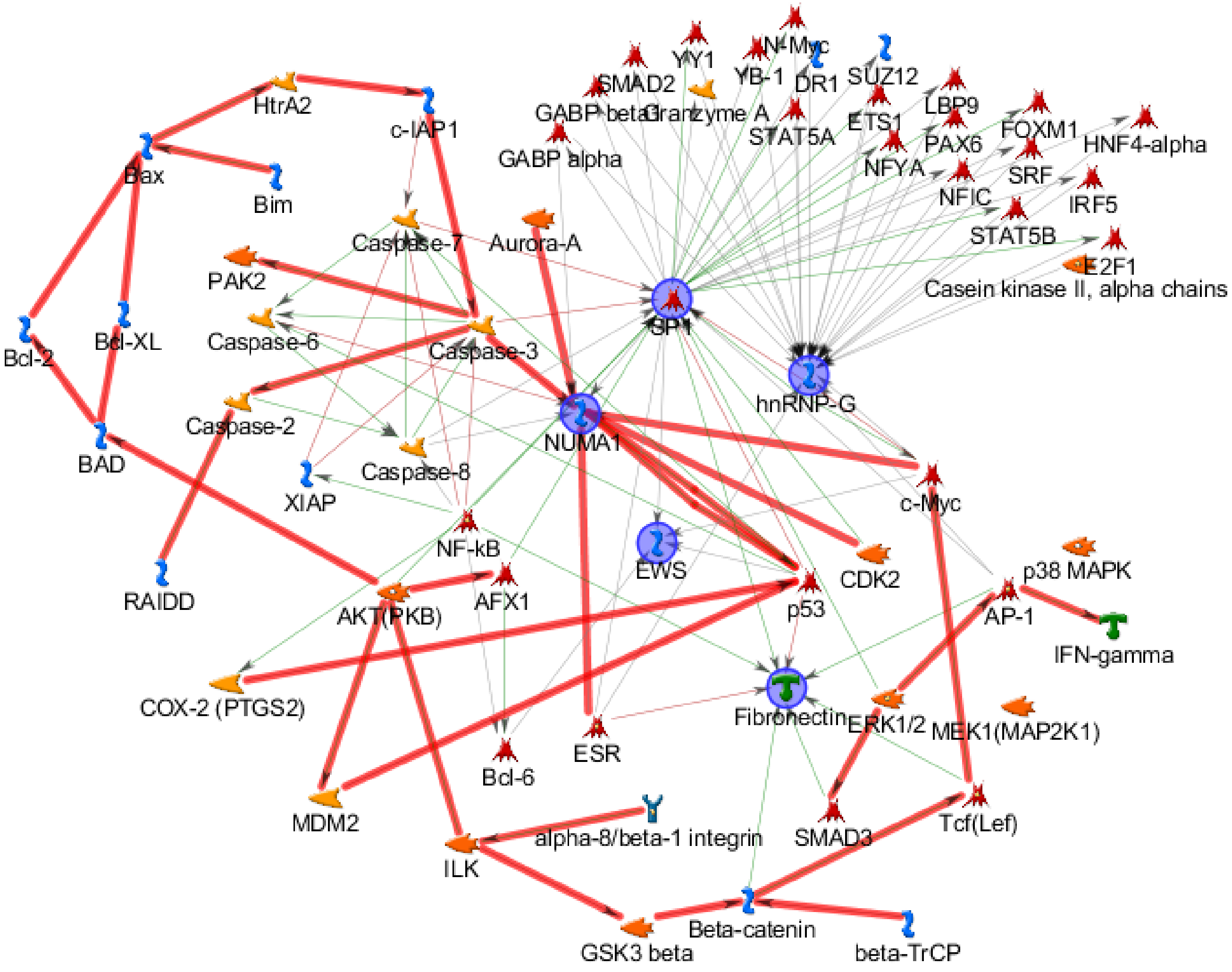

Supplementary Figure 3 The Pin1-mediated CDK1-interacting network: Red lines indicate the network composed of molecules involved in cell cycle regulation.
